# Acute effects of arabica and chicory coffee on exercise performance and post‐exercise biomarkers in young male adults: A controlled crossover study

**DOI:** 10.14814/phy2.71200

**Published:** 2026-11-08

**Authors:** Fatma Özsel Özcan, Ceyda Okudu, Hatice İlhan Odabaş, İbrahim Turgay Turan

**Affiliations:** ^1^ Department of Nutrition and Dietetics, Faculty of Health Sciences Kahramanmaraş Sütçü İmam University Kahramanmaraş Türkiye; ^2^ Department of Molecular Biology and Genetics, Faculty of Engineering and Natural Sciences Atlas University Istanbul Türkiye; ^3^ Department of Sports Management, Faculty of Sport Sciences Haliç University İstanbul Türkiye; ^4^ Department of Recreation, Faculty of Sport Sciences Haliç University İstanbul Türkiye

**Keywords:** arabica coffee, chicory coffee, exercise performance, interleukin‐6, lipoprotein lipase

## Abstract

To compare the acute effects of a single serving of Arabica and chicory coffee with a no‐coffee reference condition on exercise performance, cardiometabolic responses, and post‐exercise biomarkers in healthy young male adults. Twenty‐two moderately active young male adults aged 18–25 years completed a single‐blind controlled crossover study. The no‐coffee reference session was completed first; the Arabica and chicory coffee order was randomized with a 3‐day washout. Beverages (180 mL) were consumed 45 min before a 20‐m shuttle‐run test. Performance, cardiometabolic variables, and post‐exercise LPL, TNF‐α, adiponectin, and IL‐6 were assessed. Shuttle‐run performance, perceived exertion, and estimated VO_2_max did not differ among conditions (all *p* > 0.05). No significant condition × time interactions were observed for cardiometabolic variables. Post‐exercise LPL protein concentrations differed significantly among conditions (*p* = 0.038) and were numerically highest after chicory coffee (27.72 ng/mL). IL‐6 showed a non‐significant trend toward lower concentrations after chicory coffee (38.03 pg/mL; *p* = 0.094); TNF‐α and adiponectin did not differ significantly. Neither beverage improved acute exercise performance. Arabica and chicory coffee nevertheless showed different post‐exercise biomarker patterns that warrant confirmation in larger crossover studies.

## Graphical abstract

Effects of chicory versus Arabica coffee on exercise performance.

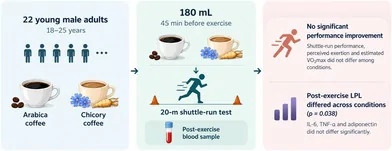

## 1 INTRODUCTION

Caffeine has the potential to affect both athletic performance and metabolic processes, and coffee is often ingested before physical activity (Guest et al., 2021; Jiménez et al., 2021). Caffeine has been shown to enhance fat oxidation during incremental exercise and improve various aspects of endurance and athletic performance, with effects dependent on the dose, habitual caffeine intake, training level and individual sensitivity (Guest et al., 2021; Ramírez‐Maldonado et al., 2021). The ergogenic effects are mainly seen at doses of 3–6 mg/kg bodyweight, which are higher than caffeine concentrations in most regular coffee servings.

Arabica coffee accounts for a large portion of the world's coffee production (International Coffee Organization, 2025) and is comprised of caffeine, chlorogenic acids and a number of other polyphenols. Their roles have been studied in the regulation of glucose, lipid metabolism, oxidative stress and adipocyte physiology (Nguyen et al., 2024). Research has shown that caffeinated coffee and drinks tend to have an ergogenic effect, but the extent of this benefit varies and appears to be affected by the dose and the habitual caffeine intake (Filip et al., 2020; Jiménez et al., 2021; Karayiğit et al., 2020). This distinction is important to the present research, as the intervention was designed to represent a standard cup of coffee as opposed to a high‐caffeine protocol specifically designed to enhance performance.

The demand for caffeine‐free coffee substitutes has increased, and chicory (*Cichorium intybus* L.) is one of the best‐known plant‐based substitutes (Löhmar & Theurillat, 2003). Chicory contains inulin, sesquiterpene lactones, flavonoids, chicoric acid and caffeoylquinic acid derivatives, and its bioactive composition is quite different from Arabica coffee (Löhmar & Theurillat, 2003; Perović et al., 2021). Empirical studies have reported that chicoric acid may affect glucose regulation (Azay‐Milhau et al., 2013). Chlorogenic acids have been associated with several pathways related to carbohydrate and lipid metabolism (Nguyen et al., 2024). Preparations from chicory have shown anti‐inflammatory effects in experimental models (Bayazid et al., 2020; Han et al., 2021). A recent randomized crossover study suggested that Brussels chicory could affect performance during exhaustive exercise and the management of lactate post‐exercise (Mao et al., 2025). However, data on the effects of chicory coffee in acute exercise situations are scarce, especially compared to traditional coffee.

Accordingly, the current study investigated the acute effects of a single dose of Arabica coffee and chicory coffee versus a no‐coffee control prior to exercise. Shuttle‐run performance, subjective exertion, cardiovascular and metabolic responses, and specific post‐exercise biomarkers were analyzed. We hypothesized that the two beverages would elicit different acute physiological and biochemical response profiles, given their contrasting caffeine and phytochemical profiles, although the effect on performance may be limited when just one serving is consumed.

## 2 MATERIALS AND METHODS

### 2.1 Study design and setting

The study was conducted as a single‐blind, controlled crossover trial at Haliç University, Istanbul. Each participant first completed a fixed no‐coffee reference/control session. The control condition was not included in the randomized sequence. The subsequent two intervention sessions consisted of Arabica coffee and chicory coffee, and the order of these two beverages was randomized at the individual level. The randomized beverage order was balanced: 11 participants received Arabica coffee first and 11 received chicory coffee first, after which each participant crossed over to the alternative beverage. A 3‐day washout period separated experimental sessions. Participants were not informed of beverage identity, although complete blinding could not be guaranteed because Arabica and chicory coffee differed in taste, aroma, and appearance; investigators were aware of beverage allocation. Exercise testing was performed in the Sports Sciences Research and Application Laboratory, and biochemical analyses were carried out in the Molecular Biology and Genetics Research Laboratory of Haliç University. Sessions were conducted under standardized morning conditions between January 1 and September 30, 2024. The study was conducted in accordance with the Declaration of Helsinki and was approved by the Biruni University Clinical Research Ethics Committee (approval no. 2015‐KAEK‐79‐23‐03). Written informed consent was obtained from all participants. The trial was retrospectively registered at ClinicalTrials.gov (NCT07094191).

### 2.2 Participants

Young male adults aged 18–25 years were recruited on a voluntary basis from students enrolled at Haliç University, Istanbul, and screened for eligibility before study enrolment. Thirty volunteers were evaluated using the Physical Activity Readiness Questionnaire for Everyone (PAR‐Q+) (Warburton et al., 2011). Eight individuals were excluded because screening identified medical or exercise‐related contraindications, including cardiovascular disease requiring medically supervised exercise, chest pain, recent syncope, an active musculoskeletal condition, type 2 diabetes with a history of hypoglycemia, or a medical condition that could interfere with safe participation. Twenty‐two participants completed the study. Lifestyle and dietary characteristics were recorded at baseline, while eligibility for the exercise protocol was determined primarily from the screening assessment and health history.

Physical activity was assessed using metabolic‐equivalent‐based criteria (Jetté et al., 1990), and all participants were classified in the moderate physical activity category.

### 2.3 Data collection and measurements

One week before the first experimental session, participants attended a one‐hour theoretical and practical familiarization session for the 20‐m shuttle‐run test. This was intended to reduce uncertainty about the procedure and minimize a possible learning effect. Participants attended each test after at least 8 h of fasting and were instructed to maintain their usual eating pattern and physical activity in the preceding days and to avoid additional caffeine‐containing foods and beverages before testing. All exercise sessions began at approximately 10:00 a.m. to reduce variability related to circadian influences on cardiovascular and metabolic responses (Scheer et al., 2010). A 3‐day washout was used between sessions, a period expected to allow caffeine clearance and recovery from the preceding maximal exercise bout (Muljadi et al., 2021). A demographic form was completed before the first trial, and 24‐h dietary records were collected before each shuttle‐run session to monitor short‐term dietary intake. The shuttle‐run test was performed on a flat, non‐slip 20‐m course marked by cones and paced by standardized audio signals. Running speed increased progressively, and the test was stopped when a participant failed twice to reach the line in time. The final completed level and lap count were recorded. Estimated VO_2_max was calculated as 3.46 × [level + (laps completed at the final level/(level × 0.4325 + 7.0048))] + 12.2 and expressed as mL·kg^−1^·min^−1^ (Mayorga‐Vega et al., 2015).

Venous blood samples were collected by a trained healthcare professional within 5 min after completion of the shuttle‐run test. Samples were centrifuged at 2000 × g for 15 min, and the plasma fraction was separated and retained until analysis. Lipoprotein lipase (LPL; manufacturer designation: LIPD, catalogue no. ELK1459), TNF‐α (catalogue no. ELK1190), adiponectin (catalogue no. ELK1233) and IL‐6 (catalogue no. ELK1156) were quantified using commercially available sandwich ELISA kits (ELK Biotechnology) according to the manufacturer's procedures. The LPL assay quantified protein concentration and did not directly measure catalytic enzyme activity. Samples and standards were analyzed in duplicate. Seven concentration standards and one blank were used for calibration; absorbance was read at 450 nm, and mean blank‐corrected absorbance values were used to construct the standard curves. The manufacturer‐specified analytical ranges were 0.63–40 ng/mL for LPL, 15.63–1000 pg/mL for TNF‐α, 7.82–500 pg/mL for IL‐6, and 0.16–10 ng/mL for adiponectin before dilution‐factor correction. Adiponectin samples were diluted 1:1000 before analysis. Concentrations were calculated from the standard curves and multiplied by the applicable dilution factor. Final results were expressed as ng/mL for LPL, pg/mL for TNF‐α and IL‐6, and μg/mL for adiponectin. All reported group concentrations fell within the corresponding analytical ranges after applying the adiponectin dilution correction.

Blood lactate was measured before exercise and within the first 5 min after exercise using a portable analyzer (Lactate Scout 4, Germany). Perceived exertion was recorded immediately after exercise using the Borg scale (Borg, 1982). Capillary blood glucose was measured before and immediately after exercise. Heart rate, systolic and diastolic blood pressure, and peripheral oxygen saturation were also assessed before and after exercise; heart rate was additionally recorded during early recovery.

### 2.4 Intervention beverages

Participants consumed 180 mL of the assigned coffee beverage 45 min before the exercise test. This volume was selected to approximate a commonly consumed single serving of coffee and to maintain practical relevance to habitual use (Mitchell et al., 2014). Chicory coffee was prepared using 20 g of roasted chicory root (*Cichorium intybus* L.) per 180 mL serving. The caffeine content of the prepared Arabica beverage was not measured analytically. Its estimated caffeine content was approximately 80–100 mg per 180 mL serving, based on reference composition data rather than a direct measurement of the study beverage. The intervention therefore represents a typical coffee exposure rather than the higher caffeine doses commonly used in ergogenic studies. A single‐serving approach was also selected to reduce the likelihood of gastrointestinal discomfort associated with larger or more concentrated coffee doses (Iriondo‐DeHond et al., 2020).

The choice of a single serving with a relatively modest estimated caffeine exposure was intentional. The aim was not to test an optimized caffeine‐supplementation protocol but to compare the acute response to beverages that could realistically be consumed before exercise. This approach improves ecological relevance, although it also means that the findings should not be interpreted as a direct test of the established ergogenic effects of 3–6 mg/kg caffeine.

Nutritional information for the chicory beverage was obtained from laboratory analyses supplied by the producer, while reference composition data for Arabica coffee were obtained from USDA FoodData Central (USDA FoodData Central, n.d.). Participant flow and the crossover sequence are shown in Figure 1.

**FIGURE 1:**
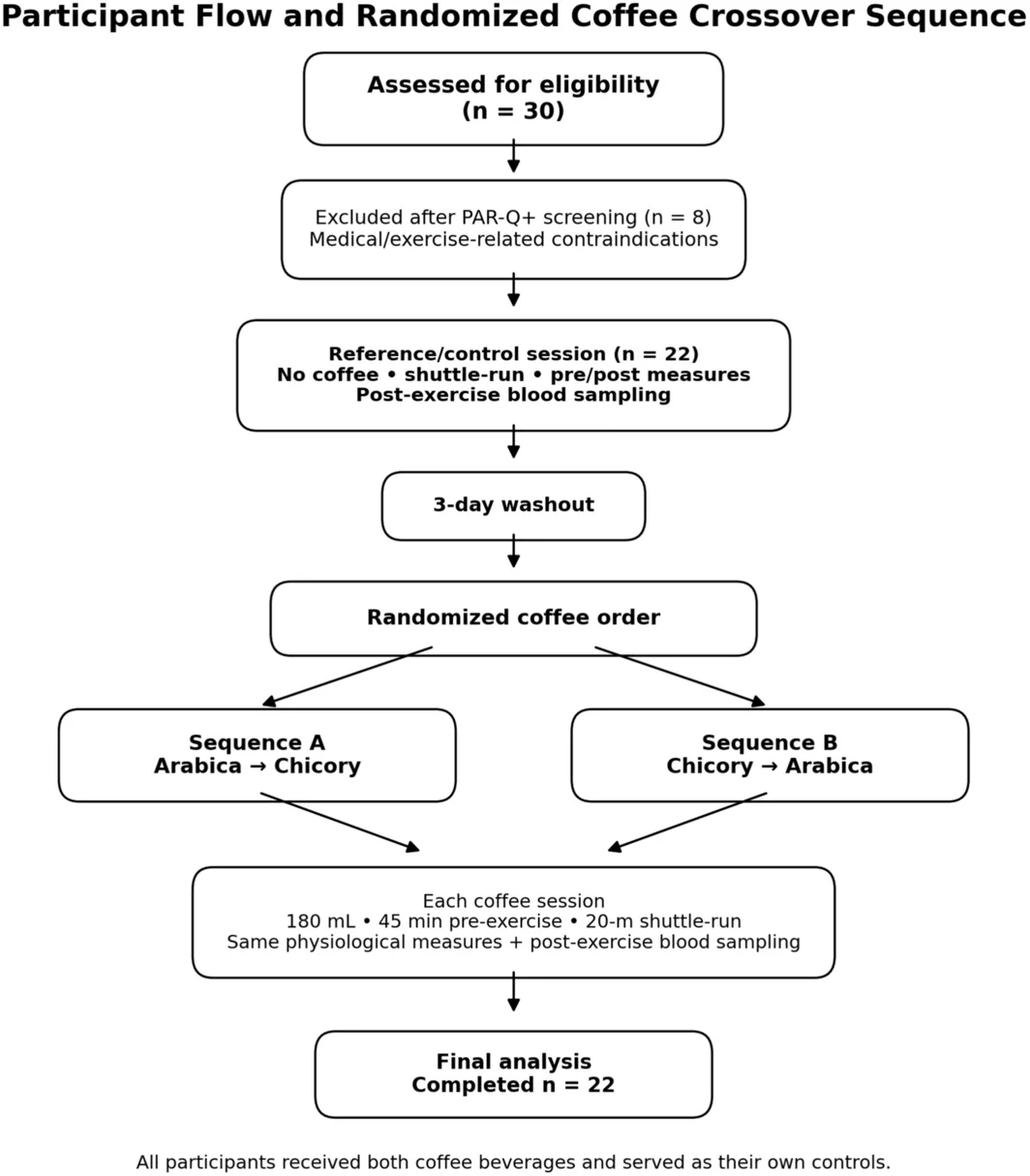
Participant flow and experimental sequence. All participants completed the fixed no‐coffee reference session first, followed by Arabica and chicory coffee sessions in randomized order with a 3‐day washout.

### 2.5 Power analysis and statistical analysis

An a priori sample size calculation was reported using G*Power version 3.1.9.7, with a medium anticipated effect size (Cohen's *f* = 0.25), a two‐sided significance level of *α* = 0.05, and a target statistical power of 80%. The effect size of *f* = 0.25 was selected based on Cohen's conventional classification of a medium effect. Given the limited availability of directly comparable studies examining the acute effects of Arabica and chicory coffee on exercise‐related outcomes, a medium effect size was considered a reasonable planning assumption rather than assuming a large intervention effect. The calculation was intended to address the condition × time interaction in the repeated‐measures design, specifically whether exercise‐induced changes differed among the no‐coffee control, Arabica coffee, and chicory coffee conditions. Each participant underwent all three conditions, with physiological outcomes assessed before and after exercise within each condition; thus, condition and time were both within‐subject factors, rather than three independent participant groups. The original calculation reported a minimum of 21 participants, and 22 participants were enrolled and completed all three conditions.

Statistical analyses were conducted using IBM SPSS Statistics version 27 (IBM Corp., Chicago, IL, USA). Continuous variables are presented as mean ± standard deviation (SD) and, where appropriate, median (range). Univariate normality was assessed using the Shapiro–Wilk test, while multivariate normality and covariance assumptions were evaluated using the Mardia and Box's *M* tests where applicable. The analyses accounted for the within‐participant dependence of observations arising from the crossover design. Participant was specified as the repeated‐measures subject, and condition (control, Arabica coffee, and chicory coffee) was treated as a within‐subject factor. Comparisons among conditions, including comparisons with the control condition, therefore accounted for the same participants completing all three conditions. For pre‐ and post‐exercise cardiometabolic outcomes, time was included as an additional within‐subject factor, and condition × time interactions were evaluated using two‐way repeated‐measures ANOVA, with Greenhouse–Geisser correction when sphericity was violated. Pillai's trace was used for multivariate inference. Within‐condition pre–post comparisons were performed using paired‐samples *t*‐tests or Wilcoxon signed‐rank tests according to distributional characteristics. Exercise‐performance outcomes across the three conditions were analyzed using one‐way repeated‐measures ANOVA when parametric assumptions were met and the Friedman test when they were not. The post‐exercise LPL, TNF‐α, IL‐6, and adiponectin panel was first evaluated using a repeated‐measures multivariate model, followed by repeated‐measures univariate analyses; non‐parametric repeated outcomes were assessed using the Friedman test. Bonferroni‐adjusted pairwise comparisons were used where applicable. Statistical significance was defined as two‐sided *p* < 0.05; values from *p* = 0.05 to *p* < 0.10 were described as statistical trends.

## 3 RESULTS

### 3.1 Participant characteristics

A total of 22 moderately active young male adults completed all study conditions and were included in the analyses. The mean age was 21.34 ± 1.98 years. At the reference assessment, mean body mass was 79.93 ± 11.97 kg and mean BMI was 25.80 ± 3.08 kg/m^2^. Anthropometric measurements remained stable across the experimental period, supporting the use of the within‐participant crossover comparisons.

### 3.2 Cardiometabolic responses

Heart rate increased significantly from pre‐ to post‐exercise and decreased during the 5‐min recovery period in all conditions. Systolic blood pressure and blood glucose also increased after exercise, while SpO_2_ showed a slight decrease. Lactate values were higher during recovery than before exercise. However, there were no significant condition × time interactions for heart rate, systolic or diastolic blood pressure, lactate, SpO_2_, or blood glucose (all *p* > 0.05). The interaction effect sizes were small (*η* ^2^ = 0.007–0.050), indicating limited differences in cardiometabolic responses among the three conditions (Table 1).

**TABLE 1:** Pre‐ and post‐exercise cardiometabolic responses across the control, Arabica coffee, and chicory coffee conditions.

Parameter	Condition	Pre‐exercise	Post‐exercise	5‐min recovery	*p*	*η* ^2^
Heart rate (beats/min)	C	83.7 ± 11.9	174.5 ± 23.6	121.6 ± 11.3	0.332	0.040
	AC	82.2 ± 13.3	165.8 ± 55.8	119.4 ± 28.5		
	CC	78.2 ± 10.4	182.9 ± 12.3	120.1 ± 12.9		
Systolic BP (mmHg)	C	117.9 ± 11.7	129.9 ± 14.9	–	0.456	0.050
	AC	114.2 ± 11.9	130.9 ± 15.9	–		
	CC	114.4 ± 9.1	121.1 ± 16.0	–		
Diastolic BP (mmHg)	C	64.9 ± 10.9	–	69.7 ± 17.9	0.723	0.010
	AC	63.8 ± 10.7	–	72.0 ± 19.7		
	CC	66.6 ± 9.9	–	63.7 ± 10.1		
Lactate (mmol/L)	C	3.2 ± 2.5	–	13.5 ± 2.1	0.764	0.007
	AC	4.1 ± 5.2	–	14.7 ± 3.1		
	CC	2.7 ± 2.5	–	13.4 ± 2.8		
SpO_2_ (%)	C	97.5 ± 1.0	96.1 ± 2.9	–	0.651	0.010
	AC	97.7 ± 0.8	96.7 ± 2.0	–		
	CC	97.5 ± 0.9	96.8 ± 2.1	–		
Blood glucose (mg/dL)	C	99.6 ± 8.8	124.8 ± 17.5	–	0.518	0.010
	AC	102.4 ± 10.5	126.7 ± 16.5	–		
	CC	99.2 ± 11.7	118.0 ± 13.9	–		

*Note*: Values are mean ± SD. *p* and *η* ^2^ represent the condition × time interaction from the two‐way repeated‐measures ANOVA with condition and time as within‐subject factors. The significance level was set at *p* < 0.05. “–” indicates not measured.

Abbreviations: AC, Arabica coffee; BP, blood pressure; C, control; CC, chicory coffee; SpO_2_, peripheral oxygen saturation.

### 3.3 Exercise performance

Exercise performance outcomes were similar across the three conditions. Neither shuttle‐run laps (*p* = 0.372), Borg perceived exertion (*p* = 0.317), nor estimated VO_2_max (*p* = 0.836) differed significantly between the control, AC, and CC conditions (Table 2).

**TABLE 2:** Exercise‐performance outcomes across the control, Arabica coffee, and chicory coffee conditions.

Outcome	Control	Arabica coffee	Chicory coffee	Test statistic	*p*
Shuttle‐run laps	54.20 ± 10.50 56 (32–74)	59.40 ± 13.80 60.5 (28–82)	56.40 ± 11.80 59.5 (30–81)	*F* = 1.006	0.372
Borg score (1–10)	7.90 ± 1.00 8 (6–10)	8.20 ± 0.80 9 (7–9)	8.20 ± 1.00 8 (6–10)	*χ* ^2^ = 2.314	0.317
Estimated VO_2_max (mL·kg^−1^·min^−1^)	38.60 ± 3.50 39 (28–46.5)	39.10 ± 3.70 40 (31–46.5)	38.70 ± 3.40 39 (29–45.9)	*χ* ^2^ = 0.319	0.836

*Note*: Model F1: 0.399; *p* = 0.878; Cohen *f* = 0.14 (shuttle‐run laps, Borg score, and estimated VO_2_max). Model F2: 0.484; *p* = 0.747; Cohen *f* = 0.12 (Borg score and estimated VO_2_max). Model F3: 0.608; *p* = 0.657; Cohen *f* = 0.14 (shuttle‐run laps and Borg score). Model F4: 0.359; *p* = 0.837; Cohen *f* = 0.11 (shuttle‐run laps and estimated VO_2_max). *F*, one‐way repeated‐measures ANOVA statistic, with condition as the within‐subject factor; *χ* ^2^, Friedman test statistic for related samples. Values are presented as mean ± SD and median (range), as applicable.

Abbreviations: AC, Arabica coffee; C, control; CC, chicory coffee.

### 3.4 Within‐condition pre–post changes

Within‐condition comparisons showed the expected acute response to the shuttle‐run test. Systolic blood pressure increased significantly in the control (*p* < 0.001), AC (*p* < 0.001), and CC conditions (*p* = 0.039), while diastolic blood pressure did not change significantly. Lactate and blood glucose increased significantly after exercise in all three conditions (all *p* < 0.001). Oxygen saturation decreased significantly from pre‐ to post‐exercise only in the AC condition (*p* = 0.023); however, the corresponding condition × time interaction was not significant (*p* = 0.651), so this finding should not be interpreted as evidence of a specific Arabica effect (Table 3).

**TABLE 3:** Within‐condition changes in cardiometabolic variables from pre‐ to post‐exercise.

Parameter	Condition	Pre‐exercise (mean ± SD)	Post‐exercise (mean ± SD)	Test statistic	*p*
Systolic BP (mmHg)	C	117.91 ± 11.72	129.91 ± 14.92	*z* = −3.842	<0.001***
	AC	114.18 ± 11.92	130.86 ± 15.89	*t* = −4.447	<0.001***
	CC	114.36 ± 9.14	121.09 ± 16.02	*t* = −2.204	0.039*
Diastolic BP (mmHg)	C	64.91 ± 10.90	69.73 ± 17.87	*z* = −0.503	0.615
	AC	63.77 ± 10.74	71.95 ± 19.70	*z* = −1.381	0.167
	CC	66.64 ± 9.87	63.73 ± 10.11	*t* = 1.460	0.159
Lactate (mmol/L)	C	3.20 ± 2.54	13.53 ± 2.08	*z* = −4.108	<0.001***
	AC	4.05 ± 5.21	14.72 ± 3.07	*z* = −4.075	<0.001***
	CC	2.71 ± 2.46	13.44 ± 2.77	*z* = −4.075	<0.001***
SpO_2_ (%)	C	97.50 ± 0.96	96.09 ± 2.86	*z* = −1.822	0.068
	AC	97.68 ± 0.78	96.68 ± 2.01	*z* = −2.273	0.023*
	CC	97.45 ± 0.86	96.82 ± 2.06	*z* = −1.084	0.278
Blood glucose (mg/dL)	C	99.55 ± 8.79	124.77 ± 17.47	*t* = −7.444	<0.001***
	AC	102.36 ± 10.54	126.68 ± 16.53	*t* = −5.737	<0.001***
	CC	99.23 ± 11.65	118.00 ± 13.88	*z* = −4.016	<0.001***

*Note*: *t*, paired‐samples *t*‐test; *z*, Wilcoxon signed‐rank test.

Abbreviations: AC, Arabica coffee; C, control; CC, chicory coffee.

* *p* < 0.05.

*** *p* < 0.001.

### 3.5 Post‐exercise biomarkers

When the four post‐exercise biomarkers were considered together, the multivariate comparison did not reach the conventional 0.05 significance level, although a trend was observed (Pillai's Trace = 0.209, *F*(8,122) = 1.779, *p* = 0.087). In the subsequent univariate analyses, LPL protein concentrations differed significantly among conditions (*F* = 3.445, *p* = 0.038) and were numerically highest after CC (27.72 ng/mL), followed by AC (19.08 ng/mL) and control (17.52 ng/mL). However, the Bonferroni‐adjusted AC–CC comparison did not reach conventional statistical significance (*p* = 0.061). IL‐6 concentrations were lowest after CC (38.03 pg/mL) and showed a statistical trend across conditions (*F* = 2.452, *p* = 0.094); the adjusted C–CC comparison was not significant (*p* = 0.140). TNF‐α (*p* = 0.267) and adiponectin (*p* = 0.581) did not differ significantly (Table 4).

**TABLE 4:** Post‐exercise absolute metabolic and inflammatory biomarker concentrations across study conditions.

Biomarker	Unit	C	AC	CC	*F*	*p*	Analytical range
LPL	ng/mL	17.52	19.08	27.72	3.445	0.038*	0.63–40
TNF‐α	pg/mL	50.25	32.75	30.25	1.351	0.267	15.63–1000
IL‐6	pg/mL	97.82	47.58	38.03	2.452	0.094†	7.82–500
Adiponectin	μg/mL	7.03	7.03	6.28	0.548	0.581	0.16–10^a^

*Note*: Values are post‐exercise concentrations. *F*, repeated‐measures ANOVA statistic; *p < 0.05; †*p* < 0.10 denotes a statistical trend. For LPL, the overall condition effect was significant (*p* = 0.038), although the Bonferroni‐adjusted AC–CC comparison did not reach conventional statistical significance (*p* = 0.061). For IL‐6, the overall condition effect represented a statistical trend (*p* = 0.094), and the adjusted C–CC comparison was not significant (*p* = 0.140). The ELISA analytical ranges were specified by the manufacturer; all reported concentrations were within range. LPL values represent ELISA‐derived protein concentrations rather than direct catalytic enzyme activity.

Abbreviations: AC, Arabica coffee; C, control; CC, chicory coffee.

^a^ The adiponectin range is shown after applying the 1:1000 dilution correction and is expressed in μg/mL.

Blood glucose increased significantly after exercise in all three conditions (all *p* < 0.001). The highest post‐exercise mean glucose value was observed after Arabica coffee (126.68 mg/dL), followed by the control condition (124.77 mg/dL) and chicory coffee (118.00 mg/dL). However, the condition × time interaction was not significant (*p* = 0.518); therefore, these numerical differences should not be interpreted as a beverage‐specific glycaemic effect (Figure 2).

**FIGURE 2:**
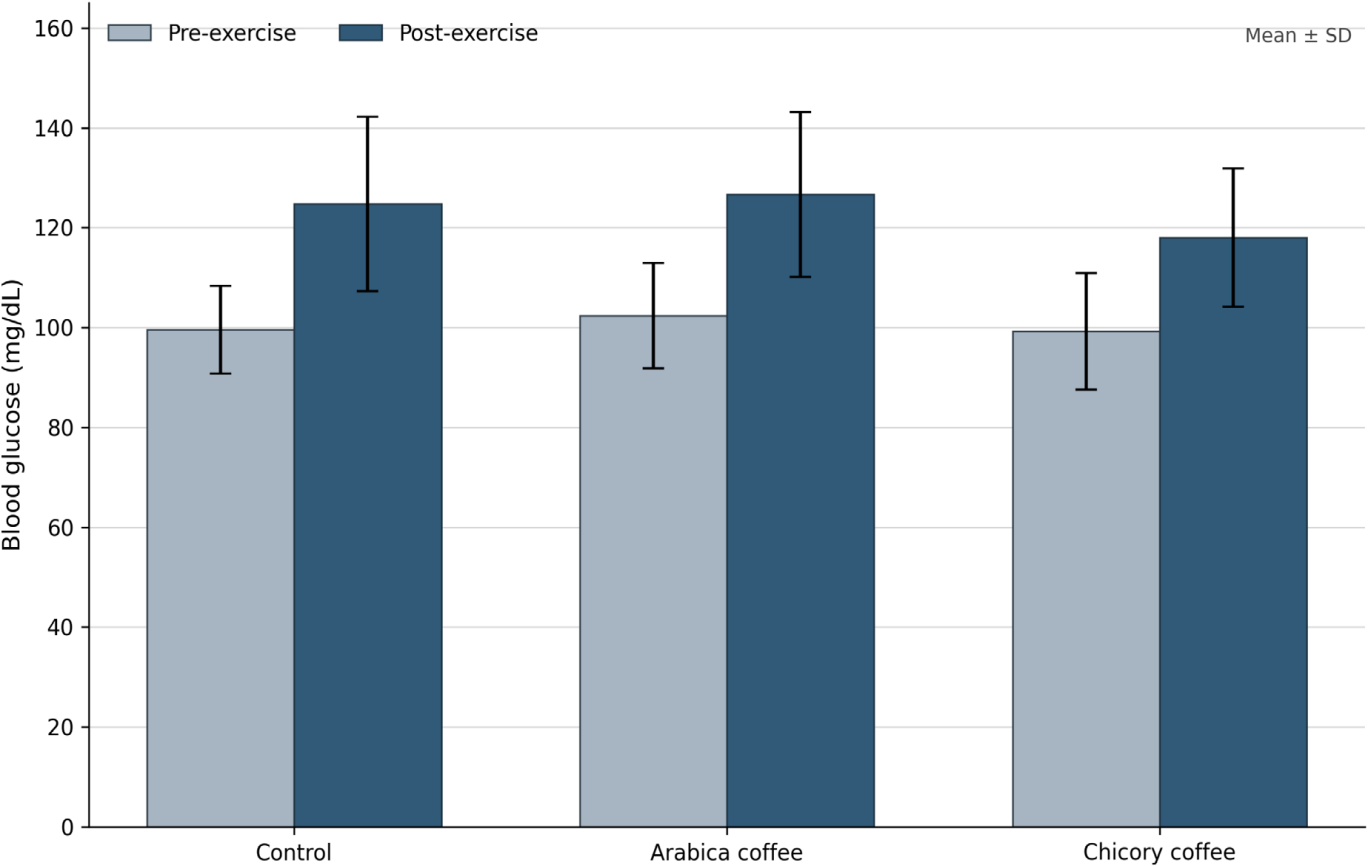
Blood glucose before and after exercise under the control, Arabica coffee, and chicory coffee conditions (*n* = 22 participants per condition). Bar heights represent means, and error bars indicate ±1 standard deviation (SD). Light bars show pre‐exercise measurements and dark bars show post‐exercise measurements. Blood glucose increased within all conditions, whereas the condition × time interaction was not significant (*p* = 0.518).

## 4 DISCUSSION

The main finding of this crossover study is that a single serving of Arabica or chicory coffee before exercise did not produce a measurable advantage in shuttle‐run performance, perceived exertion, or estimated aerobic performance in healthy young male adults. The cardiometabolic response was also dominated by the exercise bout itself: systolic blood pressure, lactate, and glucose changed substantially after exercise, but the magnitude of these changes did not differ significantly by condition. The clearest biochemical findings were a significant overall condition effect for post‐exercise LPL, with the highest absolute concentration after chicory coffee, and a non‐significant trend toward lower IL‐6 after chicory coffee. The pairwise comparisons did not reach conventional statistical significance, so these findings should be interpreted as preliminary evidence of distinct acute biomarker response patterns rather than definitive metabolic or anti‐inflammatory effects.

### 4.1 Exercise performance

The lack of a clear ergogenic effect of Arabica coffee is not unexpected when the dose used here is considered. Caffeine is a well‐established ergogenic aid, particularly for endurance exercise, but the most consistent effects are generally reported at approximately 3–6 mg/kg (Guest et al., 2021; Jiménez et al., 2021). The present intervention was closer to a habitual single cup of coffee and was estimated to provide a lower caffeine exposure than many performance‐orientated protocols; however, its caffeine content was not directly measured. Habitual caffeine intake and individual sensitivity can further modify the response (Filip et al., 2020). In addition, the 20‐m shuttle run is a progressive field test that integrates aerobic capacity, repeated acceleration and deceleration, motivation, and tolerance of increasing effort (Mayorga‐Vega et al., 2015). Borg ratings reflect the subjective experience of effort and do not necessarily change in parallel with a single objective performance measure (Borg, 1982; Houtmeyers et al., 2022). Taken together, these factors provide a plausible explanation for why the modest Arabica dose used in this study did not translate into a statistically significant performance advantage.

### 4.2 Cardiometabolic responses

Systolic blood pressure increased after exercise in all three conditions, whereas diastolic blood pressure did not change significantly. This pattern is consistent with the normal cardiovascular response to progressively increasing exercise intensity, in which cardiac output rises substantially (Mohammed et al., 2020). Caffeine can acutely modify vascular tone through antagonism of adenosine receptors and may therefore influence blood pressure in some individuals (Marcinek et al., 2024). In the present study, however, the condition × time interaction was not significant, so the larger numerical rise observed after Arabica coffee cannot be separated confidently from ordinary within‐person variability. The same caution applies to oxygen saturation. SpO_2_ decreased significantly within the Arabica condition, but the between‐condition interaction was not significant. The present data therefore do not support the stronger interpretation that Arabica coffee itself caused a clinically meaningful reduction in oxygen saturation.

Lactate increased markedly after the shuttle‐run test in every condition, confirming that the protocol imposed a substantial high‐intensity metabolic demand. A recent pilot randomized crossover study reported that Brussels chicory improved exhaustive aerobic exercise performance and post‐exercise recovery, with enhanced lactate oxidation proposed as one possible mechanism (Mao et al., 2025). Our findings did not reproduce a differential lactate response between beverages. The highest mean post‐exercise lactate value occurred after Arabica coffee, but the condition × time interaction was not significant. Differences in the form and dose of chicory, participant training status, exercise protocol, and timing of blood sampling may explain why the present results differ from that earlier study. Importantly, the current data indicate that neither coffee type materially changed the acute lactate response to maximal shuttle running.

### 4.3 Post‐exercise biomarkers

The inflammatory findings require similarly cautious interpretation. Experimental studies have shown that chicory extracts can suppress inflammatory signaling through pathways involving NF‐κB and MAPK (Bayazid et al., 2020; Han et al., 2021). Chicory seed preparations have also been investigated clinically in metabolic conditions such as non‐alcoholic fatty liver disease (Marzban et al., 2022). These observations make the lower IL‐6 values after chicory biologically interesting. Nevertheless, our overall IL‐6 comparison reached only a trend (*p* = 0.094), and the control–chicory pairwise comparison was not significant. Moreover, IL‐6 has a complex role during exercise. Contracting skeletal muscle releases IL‐6 as a myokine involved in substrate mobilization and metabolic signaling, so a lower immediate post‐exercise value should not automatically be interpreted as a beneficial suppression of inflammation (Zunner et al., 2022). Serial sampling during recovery would be necessary to determine whether chicory meaningfully changes the normal exercise‐related IL‐6 response.

LPL was the only biomarker with a statistically significant overall condition effect (*p* = 0.038). The highest absolute concentration was observed after chicory coffee (27.72 ng/mL), although the Bonferroni‐adjusted Arabica–chicory comparison remained just above the conventional significance threshold (*p* = 0.061). Lipoprotein lipase is central to triglyceride hydrolysis and tissue uptake of circulating fatty acids, and exercise itself is an important regulator of adipose‐tissue and lipid metabolism (Thompson et al., 2012). Arabica and chicory differ substantially in caffeine and phytochemical composition; chlorogenic and caffeic acids can influence AMPK‐ and PPAR‐related metabolic pathways (Vasileva et al., 2020), while caffeine has been studied for anti‐adipogenic and metabolic actions, including interactions with the intestinal microbiota (Fortunato et al., 2024). These compositional differences may have contributed to the observed LPL pattern. However, because the adjusted pairwise comparison was not significant and the assay quantified LPL protein rather than catalytic activity, the finding should not be interpreted as evidence that chicory increased LPL enzymatic activity or fat oxidation.

TNF‐α and adiponectin were not significantly different between conditions. TNF‐α is involved in the inflammatory response and muscle remodeling (Fu et al., 2024) and adiponectin is involved in insulin sensitivity, fatty acid metabolism and skeletal muscle signaling through AdipoR1 and AMPK pathways (Abou‐Samra et al., 2020). Observational studies have reported long‐term associations between habitual coffee consumption and higher adiponectin and lower inflammatory markers (Yamashita et al., 2012), but we should not assume that a single pre‐exercise dose is the cause of these long‐term associations. In all three conditions, blood glucose levels were significantly elevated after exercise. Caffeine may affect the handling of glucose during and after exercise (Fenne et al., 2023), and coffee polyphenols have been investigated in relation to glucose metabolism as a whole (Kim et al., 2025). In this experiment, there was no significant condition × time interaction for blood glucose, suggesting that the type of beverage may have had less of an effect than the acute exercise stimulus. The maintenance of circulating carbohydrate during strenuous physical activity highlights the complex interaction between carbohydrate and fat metabolism (Spriet, 2014), but the present results do not suggest a particular glycaemic advantage of either Arabica or chicory coffee.

### 4.4 Strengths, limitations, and recommendations

Several limitations should be considered. The sample was small and consisted only of moderately active young male adults, which limits generalisability to women, older adults, highly trained athletes, sedentary individuals, and clinical populations. The study evaluated only a single acute serving, and the Arabica beverage was intended to reflect normal consumption rather than a standardized high‐dose caffeine intervention. The exact caffeine and polyphenol content of the prepared beverages was not quantified analytically; the Arabica caffeine dose was estimated from reference data. In addition, the inflammatory and metabolic biomarkers were assessed at a limited post‐exercise time point; repeated sampling would have provided a clearer picture of their time course during recovery. LPL was measured by ELISA as a protein level rather than by a direct activity assay. Future studies should therefore combine repeated biomarker measurements with direct indices of lipid oxidation, such as respiratory exchange ratio, free fatty acids, glycerol, and, where possible, enzymatic LPL activity. Larger randomized crossover studies should also examine whether repeated chicory consumption over several weeks produces metabolic or recovery effects that are not detectable after a single serving. The no‐coffee reference condition was completed first in all participants, whereas only the order of the two coffee conditions was randomized. Comparisons with control may therefore be influenced by period or order effects, including learning, familiarization, and changes in motivation or recovery across visits. The familiarization session and 3‐day washout were intended to reduce these influences but cannot exclude them. Consequently, differences between a coffee condition and control cannot be attributed exclusively to the beverage intervention. In addition, the absence of a sensory‐matched placebo limited complete blinding. Despite these limitations, the within‐participant crossover structure, standardized morning testing, familiarization procedure, and repeated exposure of each participant to both coffee interventions reduce several important sources of between‐person variability.

## 5 CONCLUSIONS

In healthy, moderately active young male adults, a single serving of Arabica or chicory coffee before exercise did not significantly alter shuttle‐run performance, perceived exertion, estimated VO_2_ max, or the overall cardiometabolic response to exercise. The clearest biochemical finding was a significant overall difference in post‐exercise LPL protein concentrations (*p* = 0.038), with the highest absolute concentration after chicory coffee. Chicory coffee also showed a non‐significant tendency toward lower IL‐6 (*p* = 0.094). These findings should not be interpreted as evidence that chicory increased LPL enzymatic activity or produced a confirmed anti‐inflammatory effect. Rather, they indicate potentially different acute biochemical response patterns that merit confirmation in larger crossover studies with repeated biomarker measurements and direct measures of lipid metabolism.

## AUTHOR CONTRIBUTIONS

**Ceyda Okudu:** Conceptualization; data curation; formal analysis; funding acquisition; investigation; methodology; project administration; supervision. **Fatma Özsel Özcan:** Conceptualization; data curation; formal analysis; funding acquisition; investigation; methodology; resources; software; supervision; validation; visualization. **Hatice İlhan Odabaş:** Formal analysis; investigation; methodology; resources. **İbrahim Turgay Turan:** Data curation; formal analysis; investigation; validation.

## FUNDING INFORMATION

This research received no external funding.

## CONFLICT OF INTEREST STATEMENT

The authors declare no conflicts of interest.

## ETHICS STATEMENT

The study was conducted in accordance with the Declaration of Helsinki and was approved by the Biruni University Clinical Research Ethics Committee (approval no. 2015‐KAEK‐79‐23‐03).

## CONSENT

Written informed consent was obtained from all participants before participation.

## DECLARATION OF GENERATIVE AI USE

OpenAI's ChatGPT (GPT‐5.5 and GPT‐6; used in 2026) was used to assist with language editing, grammar and spelling corrections, refinement of academic English, sentence structure, clarity, readability, and textual coherence. AI‐assisted editorial refinements were applied primarily to the Abstract, Introduction, Materials and Methods, Discussion, and Conclusions sections. The authors directed the editing process, assessed the suggested changes against the original scientific content, and critically reviewed and approved the final wording. The study design, data collection, statistical analyses, and scientific interpretations remained the responsibility of the authors. The authors take full responsibility for the accuracy, originality, and integrity of the manuscript. No copyright or ownership rights in the manuscript were transferred to the AI technology provider.

## Data Availability

De‐identified participant‐level data may be made available by the corresponding author upon reasonable request, subject to ethics and institutional requirements.

## References

[phy271200-bib-0001] Abou‐Samra, M. , Selvais, C. M. , Dubuisson, N. , & Brichard, S. M. (2020). Adiponectin and its mimics on skeletal muscle: Insulin sensitizers, fat burners, exercise mimickers, muscling pills, or everything together? International Journal of Molecular Sciences, 21, 2620. 10.3390/ijms21072620 PMC7178193 32283840

[phy271200-bib-0002] Azay‐Milhau, J. , Ferrare, K. , Leroy, J. , Aubaterre, J. , Tournier, M. , Lajoix, A. D. , & Tousch, D. (2013). Antihyperglycemic effect of a natural chicoric acid extract of chicory (*Cichorium intybus* L.): A comparative in vitro study with the effects of caffeic and ferulic acids. Journal of Ethnopharmacology, 150, 755–760. 10.1016/j.jep.2013.09.046 24126061

[phy271200-bib-0003] Bayazid, A. B. , Park, S. H. , Kim, J. G. , & Lim, B. O. (2020). Green chicory leaf extract exerts anti‐inflammatory effects by suppressing LPS‐induced MAPK/NF‐κB activation and exhibits hepatoprotective activity in vitro. Food and Agricultural Immunology, 31, 513–532. 10.1080/09540105.2020.1742667

[phy271200-bib-0004] Borg, G. A. V. (1982). Psychophysical bases of perceived exertion. Medicine and Science in Sports and Exercise, 14, 377–381. 7154893

[phy271200-bib-0005] Fenne, K. T. , Clauss, M. , Schäfer Olstad, D. , Johansen, E. I. , & Jensen, J. (2023). An acute bout of endurance exercise does not prevent the inhibitory effect of caffeine on glucose tolerance the following morning. Nutrients, 15(8), 1941. 10.3390/nu15081941 PMC10143402 37111160

[phy271200-bib-0006] Filip, A. , Wilk, M. , Krzysztofik, M. , & del Coso, J. (2020). Inconsistency in the ergogenic effect of caffeine in athletes who regularly consume caffeine: Is it due to the disparity in the criteria that defines habitual caffeine intake? Nutrients, 12(4), 1087. 10.3390/nu12041087 PMC7230656 32326386

[phy271200-bib-0007] Fortunato, I. M. , Pereira, Q. C. , Oliveira, F. S. , Alvarez, M. C. , Santos, T. W. D. , & Ribeiro, M. L. (2024). Metabolic insights into caffeine's anti‐adipogenic effects: An exploration through intestinal microbiota modulation in obesity. International Journal of Molecular Sciences, 25(3), 1803. 10.3390/ijms25031803 PMC10855966 38339081

[phy271200-bib-0008] Fu, Y. , Nie, J. R. , Shang, P. , Zhang, B. , Yan, D. W. , Hao, X. , & Zhang, H. (2024). Tumor necrosis factor alpha deficiency promotes myogenesis and muscle regeneration. Zoological Research, 45, 951–960. 10.24272/j.issn.2095-8137.2024.039 PMC11298682 39021083

[phy271200-bib-0009] Guest, N. S. , VanDusseldorp, T. A. , Nelson, M. T. , Grgic, J. , Schoenfeld, B. J. , Jenkins, N. D. M. , Arent, S. M. , Antonio, J. , Stout, J. R. , Trexler, E. T. , Smith‐Ryan, A. E. , Goldstein, E. R. , Kalman, D. S. , & Campbell, B. I. (2021). International society of sports nutrition position stand: Caffeine and exercise performance. Journal of the International Society of Sports Nutrition, 18(1), 1. 10.1186/s12970-020-00383-4 PMC7777221 33388079

[phy271200-bib-0010] Han, C. , Wu, X. , Zou, N. , Zhang, Y. , Yuan, J. , Chen, W. , Gao, Y. , Yao, J. , Li, C. , Hou, J. , & Qin, D. (2021). Cichorium pumilum Jacq extract inhibits LPS‐induced inflammation via MAPK signalling pathway and protects rats from hepatic fibrosis caused by abnormalities in the gut‐liver axis. Frontiers in Pharmacology, 12, 683613. 10.3389/fphar.2021.683613 PMC8117150 33995112

[phy271200-bib-0011] Houtmeyers, K. , Lenoir, M. , de Beukelaer, N. , Goudsmit, J. , & Geraets, L. (2022). Differential ratings of perceived exertion: Relationships with external intensity and load in elite men's football. Frontiers in Physiology, 13, 915481. 10.3389/fphys.2022.915481 35661057

[phy271200-bib-0012] International Coffee Organization . (2025). Coffee market report. International Coffee Organization.

[phy271200-bib-0013] Iriondo‐DeHond, A. , Uranga, J. A. , Del Castillo, M. D. , & Abalo, R. (2020). Effects of coffee and its components on the gastrointestinal tract and the brain‐gut axis. Nutrients, 13(1), 88. 10.3390/nu13010088 PMC7824117 33383958

[phy271200-bib-0014] Jetté, M. , Sidney, K. , & Blümchen, G. (1990). Metabolic equivalents (METS) in exercise testing, exercise prescription, and evaluation of functional capacity. Clinical Cardiology, 13, 555–565. 10.1002/clc.4960130809 2204507

[phy271200-bib-0015] Jiménez, S. L. , Díaz‐Lara, J. , Pareja‐Galeano, H. , & del Coso, J. (2021). Caffeinated drinks and physical performance in sport: A systematic review. Nutrients, 13(9), 2944. 10.3390/nu13092944 PMC8466927 34578821

[phy271200-bib-0016] Karayiğit, R. , Naderi, A. , Akca, F. , Cruz, C. J. G. , Sarshin, A. , Yasli, B. C. , Ersoz, G. , & Kaviani, M. (2020). Effects of different doses of caffeinated coffee on muscular endurance, cognitive performance, and cardiac autonomic modulation in caffeine naive female athletes. Nutrients, 13(1), 2. 10.3390/nu13010002 PMC7821939 33374947

[phy271200-bib-0017] Kim, H. , Kim, S. R. , & Jung, U. J. (2025). Coffee and its major polyphenols in the prevention and management of type 2 diabetes: A comprehensive review. International Journal of Molecular Sciences, 26, 5544. 10.3390/ijms26125544 PMC12193296 40565007

[phy271200-bib-0018] Löhmar, K. , & Theurillat, V. (2003). Chicory beverages. In B. Caballero , P. Finglas , & F. Toldrá (Eds.), Encyclopedia of food sciences and nutrition (Vol. 2003, 2nd ed., pp. 1144–1149). Academic Press. 10.1016/B0-12-227055-X/00210-8

[phy271200-bib-0019] Mao, Y. , Huang, J. , Li, S. , Chen, G. , Du, Y. , Kang, M. , Zhu, S. , Zhang, W. , Xu, Q. , Wang, Y. , Ling, W. , Luo, X. , & Wang, D. (2025). Brussels chicory enhances exhaustive aerobic exercise performance and post‐exercise recovery, possibly through promotion of lactate oxidation: A pilot randomized, single‐blind, placebo‐controlled, two‐way crossover study. Nutrients, 17, 365. 10.3390/nu17020365 PMC11769108 39861495

[phy271200-bib-0020] Marcinek, K. , Luzak, B. , & Rozalski, M. (2024). The effects of caffeine on blood platelets and the cardiovascular system through adenosine receptors. International Journal of Molecular Sciences, 25, 8905. 10.3390/ijms25168905 PMC11354695 39201591

[phy271200-bib-0021] Marzban, M. , Bahrami, M. , Kamalinejad, M. , Tahamtan, M. , Khavasi, N. , Hajimonfarednejad, M. , & Jameshorani, M. (2022). The therapeutic effects of chicory seed aqueous extract on cardio‐metabolic profile and liver enzymes in nonalcoholic fatty liver disease: A double‐blind randomized clinical trial. Immunopathology Perspectives, 8, e28262. 10.34172/ipp.2022.28262

[phy271200-bib-0022] Mayorga‐Vega, D. , Aguilar‐Soto, P. , & Viciana, J. (2015). Criterion‐related validity of the 20‐m shuttle run test for estimating cardiorespiratory fitness: A meta‐analysis. Journal of Sports Science and Medicine, 14, 536–547. PMC4541117 26336340

[phy271200-bib-0023] Mitchell, D. C. , Knight, C. A. , Hockenberry, J. , Teplansky, R. , & Hartman, T. J. (2014). Beverage caffeine intakes in the U.S. Food and Chemical Toxicology, 63, 136–142. 10.1016/j.fct.2013.10.042 24189158

[phy271200-bib-0024] Mohammed, L. L. M. , Dhavale, M. , Abdelaal, M. K. , Alam, A. B. M. N. , Blazin, T. , Prajapati, D. , & Mostafa, J. A. (2020). Exercise‐induced hypertension in healthy individuals and athletes: Is it an alarming sign? Cureus, 12, e11988. 10.7759/cureus.11988 PMC7793423 33437543

[phy271200-bib-0025] Muljadi, J. A. , Kaewphongsri, P. , Chaijenkij, K. , Gaysonsiri, D. , & Prasertsri, P. (2021). Effect of caffeine on delayed‐onset muscle soreness: A meta‐analysis of randomized controlled trials. Bulletin of the National Research Centre, 45, 197. 10.1186/s42269-021-00660-5

[phy271200-bib-0026] Nguyen, V. , Taine, E. G. , Meng, D. , Cui, T. , & Tan, W. (2024). Chlorogenic acid: A systematic review on the biological functions, mechanistic actions, and therapeutic potentials. Nutrients, 16(7), 924. 10.3390/nu16070924 PMC11013850 38612964

[phy271200-bib-0027] Perović, J. , Tumbas Šaponjac, V. , Kojić, J. , Krulj, J. , Moreno, D. A. , García‐Viguera, C. , Bodroža‐Solarov, M. , & Ilić, N. (2021). Chicory (*Cichorium intybus* L.) as a food ingredient ‐ nutritional composition, bioactivity, safety, and health claims: A review. Food Chemistry, 336, 127676. 10.1016/j.foodchem.2020.127676 32768902

[phy271200-bib-0028] Ramírez‐Maldonado, M. , Jurado‐Fasoli, L. , del Coso, J. , Ruiz, J. R. , & Amaro‐Gahete, F. J. (2021). Caffeine increases maximal fat oxidation during a graded exercise test: Is there a diurnal variation? Journal of the International Society of Sports Nutrition, 18(1), 5. 10.1186/s12970-020-00400-6 PMC7792284 33413459

[phy271200-bib-0029] Scheer, F. A. J. L. , Hu, K. , Evoniuk, H. , Kelly, E. E. , Malhotra, A. , Hilton, M. F. , & Shea, S. A. (2010). Impact of the human circadian system, exercise, and their interaction on cardiovascular function. Proceedings of the National Academy of Sciences of the United States of America, 107(47), 20541–20546. 10.1073/pnas.1006749107 PMC2996667 21059915

[phy271200-bib-0030] Spriet, L. L. (2014). New insights into the interaction of carbohydrate and fat metabolism during exercise. Sports Medicine, 44(Suppl 1), S87–S96. 10.1007/s40279-014-0154-1 PMC4008806 24791920

[phy271200-bib-0031] Thompson, D. , Karpe, F. , Lafontan, M. , & Frayn, K. (2012). Physical activity and exercise in the regulation of human adipose tissue physiology. Physiological Reviews, 92, 157–191. 10.1152/physrev.00012.2011 22298655

[phy271200-bib-0032] USDA FoodData Central . (n.d.). U.S. Department of Agriculture. https://fdc.nal.usda.gov

[phy271200-bib-0033] Vasileva, L. V. , Savova, M. S. , Amirova, K. M. , Balcheva‐Sivenova, Z. , Ferrante, C. , Orlando, G. , Wabitsch, M. , & Georgiev, M. I. (2020). Caffeic and chlorogenic acids synergistically activate browning program in human adipocytes: Implications of AMPK‐ and PPAR‐mediated pathways. International Journal of Molecular Sciences, 21, 9740. 10.3390/ijms21249740 PMC7766967 33371201

[phy271200-bib-0034] Warburton, D. E. R. , Jamnik, V. K. , Bredin, S. S. D. , & Gledhill, N. (2011). The physical activity readiness questionnaire for everyone (PAR‐Q+) and electronic physical activity readiness medical examination (ePARmed‐X+). Health & Fitness Journal of Canada, 4, 3–23. 10.14288/hfjc.v4i2.103

[phy271200-bib-0035] Yamashita, K. , Yatsuya, H. , Muramatsu, T. , Toyoshima, H. , Murohara, T. , & Tamakoshi, K. (2012). Association of coffee consumption with serum adiponectin, leptin, inflammation and metabolic markers in Japanese workers: A cross‐sectional study. Nutrition & Diabetes, 2, e33. 10.1038/nutd.2012.6 PMC3347755 23169586

[phy271200-bib-0036] Zunner, B. E. M. , Wachsmuth, N. B. , Eckstein, M. L. , Scherl, L. , Schierbauer, J. R. , Haupt, S. , Stumpf, C. , Reusch, L. , & Moser, O. (2022). Myokines and resistance training: A narrative review. International Journal of Molecular Sciences, 23, 3501. 10.3390/ijms23073501 PMC8998961 35408868

